# Thioredoxin Modulates Protein Arginine Deiminase 4 (PAD4)-Catalyzed Citrullination

**DOI:** 10.3389/fimmu.2019.00244

**Published:** 2019-02-19

**Authors:** Mitesh Nagar, Ronak Tilvawala, Paul R. Thompson

**Affiliations:** ^1^Department of Biochemistry and Molecular Pharmacology, University of Massachusetts Medical School, Worcester, MA, United States; ^2^Program in Chemical Biology, University of Massachusetts Medical School, Worcester, MA, United States

**Keywords:** thioredoxin, PAD4, citrullination, rheumatoid arthritis, autoantibodies, NETosis

## Abstract

Protein citrullination is a post-translational modification catalyzed by the protein arginine deiminases (PADs). This modification plays a crucial role in the pathophysiology of numerous autoimmune disorders including RA. Recently, there has been a growing interest in investigating physiological regulators of PAD activity to understand the primary cause of the associated disorders. Apart from calcium, it is well-documented that a reducing environment activates the PADs. Although the concentration of thioredoxin (hTRX), an oxidoreductase that maintains the cellular reducing environment, is elevated in RA patients, its contribution toward RA progression or PAD activity has not been explored. Herein, we demonstrate that hTRX activates PAD4. Kinetic characterization of PAD4 using hTRX as the reducing agent yielded parameters that are comparable to those obtained with a routinely used non-physiological reducing agent, e.g., DTT, suggesting the importance of hTRX in PAD regulation under physiological conditions. Furthermore, we show that various hTRX mutants, including redox inactive hTRX variants, are capable of activating PAD4. This indicates a mechanism that does not require oxidoreductase activity. Indeed, we observed non-covalent interactions between PAD4 and hTRX variants, and propose that these redox-independent interactions are sufficient for hTRX-mediated PAD4 activation.

## Introduction

Protein citrullination is a post-translational modification that converts a positively charged arginine residue into a neutral citrulline (1, 2). Modifications of such charged residues can alter various protein properties, including protein-protein interactions and protein-DNA interactions, with consequent effects on various physiological processes (2–4). For example, histone hyper-citrullination leads to chromatin decondensation and ultimately the release of neutrophil extracellular traps (NETs), which are involved in the innate immune response against various pathogens as well as in the pathogenesis of autoimmune and inflammatory disorders (5–7). In fact, protein citrullination has been extensively studied in the context of rheumatoid arthritis (RA) due to the presence of autoantibodies against citrullinated proteins (ACPA) in over 70% of RA patients (3, 8–10). ACPA are highly specific and widely used for the diagnosis of RA (11, 12). Apart from autoimmune diseases, like RA, lupus, multiple sclerosis, and ulcerative colitis (13–15), aberrant citrullination has also been shown to play a role in the pathology of various neurodegenerative diseases, diabetes, and cancer (16–19). Therefore, the group of enzymes that catalyze protein citrullination, the protein arginine deiminases (PADs), are attractive therapeutic targets (13, 14, 20–24). For this reason, most PAD-focused studies investigate their pathophysiological functions rather than their physiological roles under normal circumstances. Importantly, it is still unclear how PAD activity is regulated and how aberrant regulation leads to various pathological conditions.

There are five human PAD isozymes (PAD1-4 and PAD6). These isozymes are localized in specific tissues to carry out distinct physiological functions. For example, PAD1 and PAD3 are expressed in the skin and hair follicles and regulate the cellular architecture. PAD2 and PAD4 play roles in apoptosis, gene regulation, and NET-formation (2, 6, 25–27). Amongst the PAD family, PAD2 and PAD4 are of particular interest because these isozymes are expressed mainly in immune cells and have been implicated in a variety of autoimmune and inflammatory disorders (3, 13–15, 28, 29). All the active PADs (PAD1-4) share similar catalytic mechanisms and their activity is regulated by calcium through important conformational changes that facilitate PAD-catalysis (30–32). In addition to calcium, bicarbonate and GSH regulate PAD2 and PAD4 activity under physiological conditions (33, 34). Since redox balance is a necessary requirement for PAD activity, thioredoxin (hTRX), a 12 kDa protein that regulates redox homoeostasis in all tissues of the human body (35–37), is another candidate that could impact PAD-catalyzed citrullination. In fact, numerous studies have shown elevated levels of hTRX in serum and synovial samples from RA patients (38–42) suggesting hTRX activity might contribute to RA progression; however its role in PAD activation has yet been established.

Herein, we report the impact of hTRX on PAD activity using the synthetic substrate mimic *N*_α_-benzoyl-L-arginine ethyl ester (BAEE) and verified our results with the physiological substrate histone H3. Moreover, we used a variety of hTRX mutants to show that hTRX activates PAD4 in a redox-independent manner.

## Materials and Methods

### Plasmids

His-tagged human wild-type hTRX and C35S, C32/35S, C62S, C69S, and C73S-hTRX variants were kind gifts from Dr. Weerapana (Boston College, MA). The C32/35/69S and C62/69S-hTRX mutants were created in house.

### Preparation of Proteins

#### Thioredoxins

Wild-type hTRX and its mutants were purified in the absence of any reducing agents as described previously (43). All variants were fully reduced with DTT (10 mM) for 10 min at 37°C (44) and the excess DTT was removed by exhaustive buffer exchange using amicon centrifugal filters (3 kDa).

#### PAD4

Purification of human PAD4 was carried out as described previously (45) but without reducing agent. *S*-nitrosoglutathione (GSNO) and H_2_O_2_-modified PAD4 were prepared by incubating PAD4 with GSNO (200 μM) or H_2_O_2_ (100 μM) for 10 min at 37°C. Excess reagents were removed by exhaustive buffer exchange using amicon centrifugal filters (10 kDa).

### PAD4 Activity Assay

#### Using BAEE as Substrate

PAD4 activation was determined using a previously described discontinuous colorimetric assay (45). Briefly, reaction buffer (Tris-HCl, 100 mM; pH 7.6; NaCl, 50 mM) containing CaCl_2_ (10 mM), BAEE (10 mM), and hTRX (0–10 μM) was pre-incubated at 37°C for 10 min followed by the addition of PAD4 (final concentration of 0.2 μM) to initiate the reaction (60 μL final volume). After 10 min, reactions were quenched by adding 200 μL of freshly prepared COLDER solution [H_3_PO_4_, 2.25 M; H_2_SO_4_, 4.5 M; NH_4_Fe(SO_4_), 1.5 mM; diacetyl monoxime, 20 mM; and thiosemicarbazide, 1.5 mM] and incubated for 30 min at 95°C for color development. Sample absorbance was measured at 540 nm to determine the amount citrulline produced during the course of the reaction using a citrulline standard. Control activity of PAD4 was measured using a saturating concentration of DTT (2 mM) (45).

#### Using Histone H3 as Substrate

Previously purified Histone H3 (10 μM) (46) was treated with PAD4 (0.2 μM) in reaction buffer containing thioredoxin (0. 0.08, 0.17, 0.33, 0.67, 1.33, and 2.67 μM) at 37°C (total volume 30 μL) for 10 min and then the reaction was quenched using 6X SDS-PAGE loading dye followed by 10 min incubation at 95°C before running samples on SDS-PAGE. Separated proteins were then transferred to PVDF membrane (Bio-Rad) at 80 V for 50 min. The membrane was then blocked with PBS containing Tween-20 (0.1%, PBST) and BSA (5%) for 1 h at room temperature before adding primary antibodies for PAD4 (rabbit, 1:1,000, Proteintech cat# 7373-1-AP), histone H3 (mouse, 1:1,000, Abcam cat# ab10799), and histone H3-Cit-2,8,17 (rabbit, 1:1,000, Abcam cat# ab5103) and further incubated overnight at 4°C. The next day, after washing with PBST (3×), the membrane was incubated with secondary anti-rabbit IgG Licor conjugate antibody (1:5,000) for 1 h at room temperature. Finally, the membrane was imaged and quantified using LICOR Odyssey Imaging System.

### hTRX Activity Assay

The hTRX-activity assay was performed as per the manufacturer's instructions (Cayman Chemical Co., cat# 20039). Briefly, various hTRX variant samples (10 μL of 1.0 μM solution) were added to a 96-well half area black plate followed by addition of assay buffer (55 μL), human thioredoxin reductase (10 μL of 1.0 μM solution), and NADPH (5 μL, diluted according to kit instructions) to each sample well. The plate was then incubated at 37°C for 30 min. After incubation, fluorescent substrate (20 μL, diluted according to kit instructions) was added to each sample as quickly as possible. The plate was immediately placed in an Envision plate reader (Molecular Devices) and fluorescence was monitored at 480 nm excitation and 520 nm emission for 15 min. Reaction rates were plotted and compared.

### hTRX Interaction With PAD4

#### Size-Exclusion Chromatography

PAD4 (12 μM) and hTRX (or its variants, 80 μM) were incubated in size-exclusion buffer (SEC-buffer, Tris-Cl, 100 mM; pH 7.6; NaCl, 50 mM, and CaCl_2_, 10 mM) at 37°C for 10 min (final volume 100 μL). This mixture was diluted with 300 μL of SEC-buffer and loaded onto a size exclusion column (Superdex 200 10/300 GL, GE Healthcare) attached to an ÄKTA-FPLC. The proteins were separated with a flow rate of 0.5 mL/min. Peak fractions (1 mL) corresponding to the PAD4 dimer and PAD4 monomer were concentrated 10X and subjected to denaturing SDS-PAGE followed by blotting on PVDF membrane (see above). Primary antibodies against PAD4 (rabbit, 1:1,000, Proteintech cat# 7373-1-AP) and thioredoxin (mouse, 1:1,000, Abcam cat# ab16965) were used to evaluate the presence of hTRX in PAD4 peaks.

#### Immunoprecipitation (IP)

HL60 cells were grown in RPMI 1640 medium (w/L-glutamine and HEPES) supplemented with 10% FBS and penicillin/streptomycin at a density of 3 × 10^5^ cells/mL. Differentiation of HL60 cells to granulocytes was induced by 1.3% dimethyl sulfoxide (DMSO) and then the cells were allowed to grow for 4 days before harvesting. After washing the cell pellet with PBS (3×), cells were lysed using lysis buffer (Tris-Cl, 25 mM, pH 7.4; NaCl, 50 mM; CaCl_2_, 2 mM and 1% NP40) containing protease inhibitor cocktail. Clarified cell lysates (1.2 mg total protein) were incubated with anti-PAD4 (4 μg, rabbit, Proteintech cat# 7373-1-AP) or generic rabbit IgG (4 μg) for overnight at 4°C with gentle rotation. Protein A/G plus-agarose beads (100 μL slurry, Santa Cruz Biotechnology) were added to the mixture and incubated for another 4 h at 4°C to capture antibody-bound protein complexes. The beads were then washed (3×) with wash buffer [Tris-Cl, 25 mM (pH 7.4); NaCl, 50 mM, CaCl_2_, 2 mM; and 0.2% NP-40], and the protein complexes were eluted using 2 × SDS sample buffer. Eluted proteins were immunoblotted as mentioned above using primary antibodies against PAD4 (mouse 1:1,000, Abcam cat# 128086) and thioredoxin (mouse, 1:1,000, Abcam cat# ab16965). HRP-conjugated goat anti-mouse antibody (1:10,000, Invitrogen cat # 31432) was used to evaluate the presence of hTRX and PAD4 in the elution fraction by detecting chemiluminescence using an ECL kit (GE Healthcare Amersham).

## Results

### Effect of hTRX on PAD4 Activity

**Figure 1:**
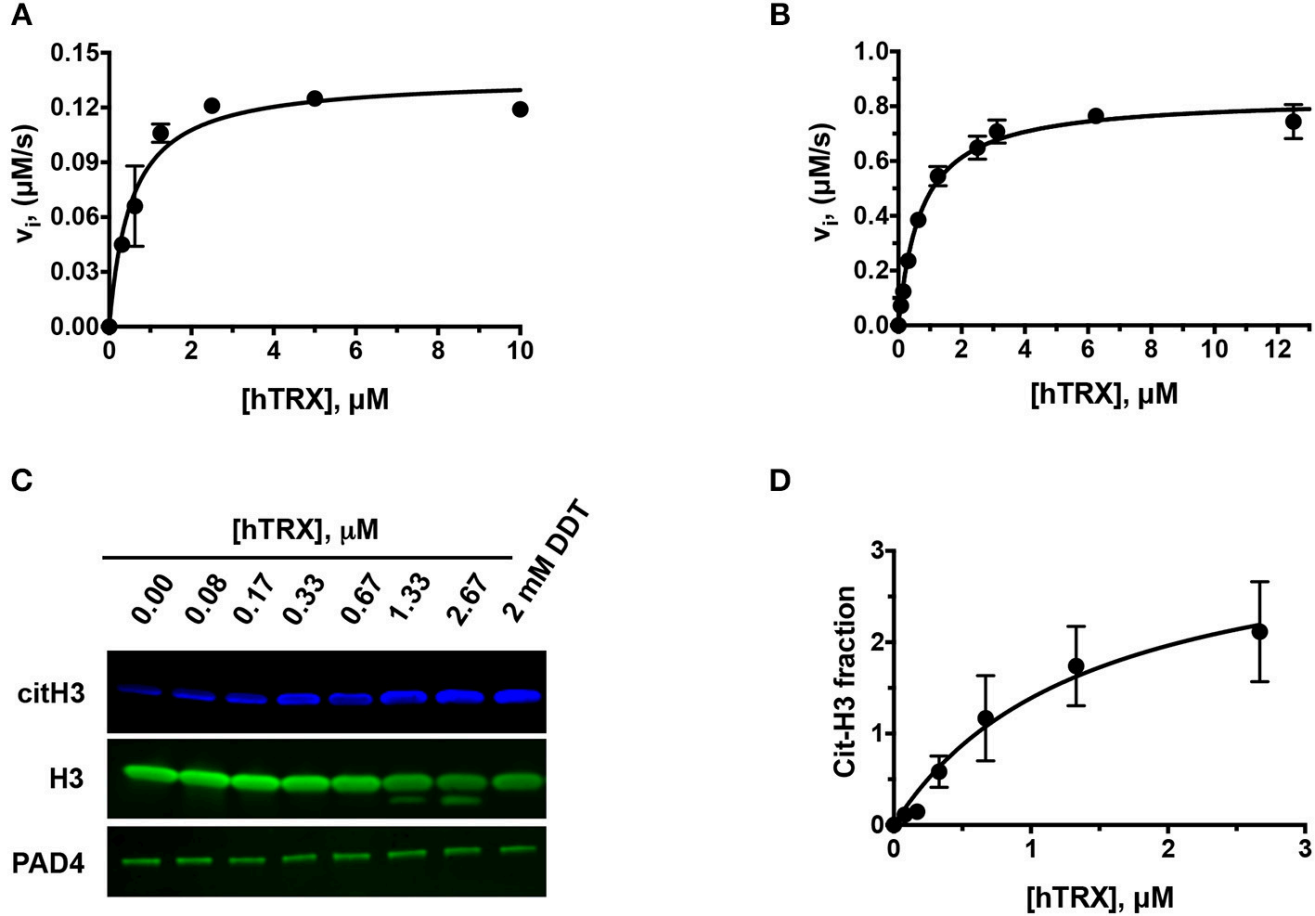
Effect of hTRX on PAD4 activity. Rate of citrullination of BAEE by PAD4 in presence of various concentrations of untreated **(A)** and DTT-treated hTRX **(B)**. Histone H3 citrullination by PAD4 in the presence of various hTRX concentrations **(C)**. The quantification of the western blot, i.e., citH3/H3 ratio, with increasing concentration of hTRX **(D)**.

To investigate whether hTRX can influence PAD4 activity, we used BAEE (Figures 1A,B) and histone H3 as substrates (Figures 1C,D). Purified hTRX activated PAD4 in a concentration-dependent manner and displayed saturation kinetics (Figures 1A,B). Although DTT-treated hTRX showed better activation (Figure 1B), maximal PAD4 activity was still observed at 5 μM of hTRX (Figures 1A,B). Therefore, all other kinetic assays were performed using this saturating concentration of reduced-hTRX.

### Activation of PAD4 With Various Reducing Agents

**Figure 2:**
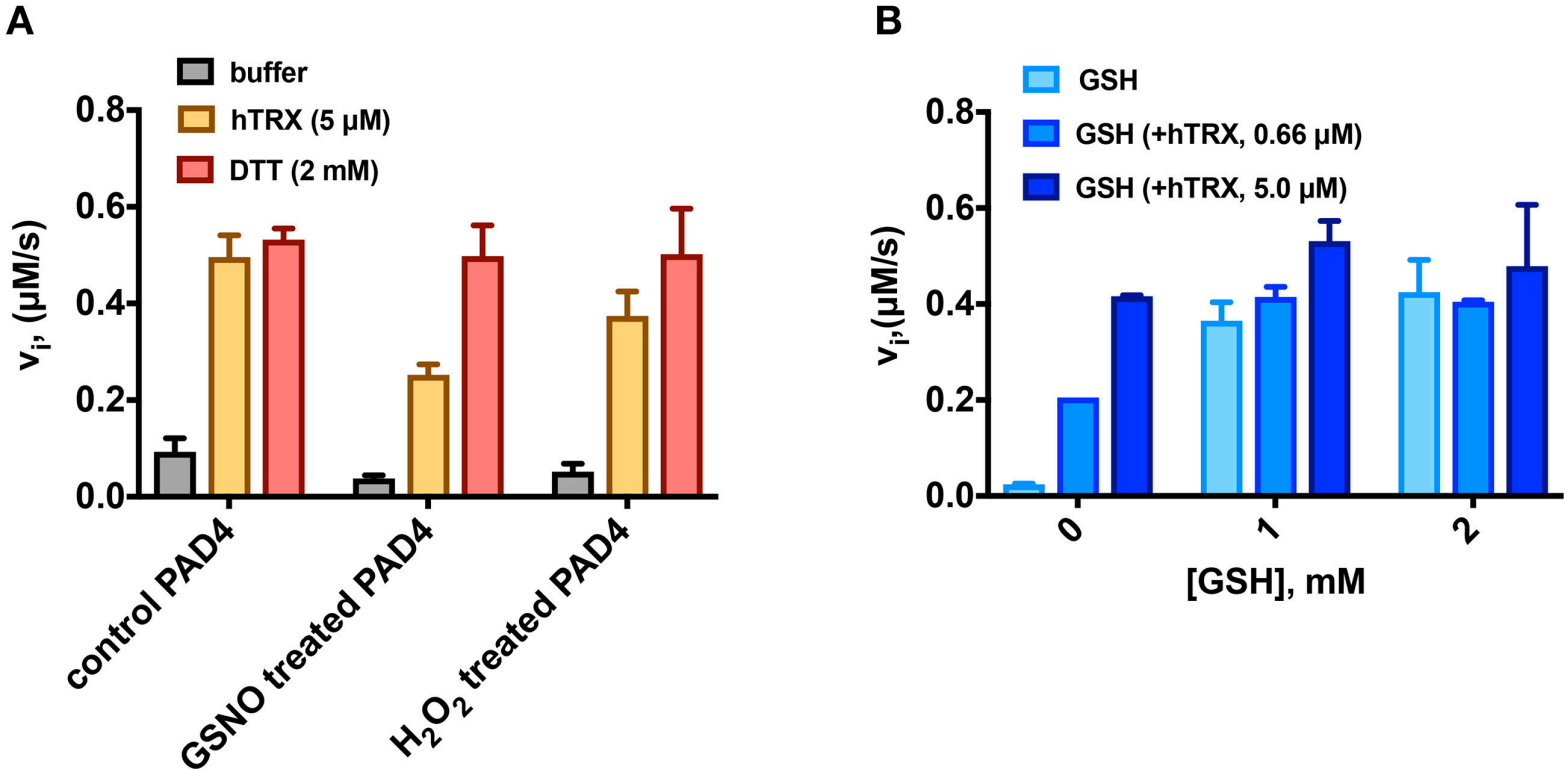
Activation of PAD4 in the presence of various reducing agents. **(A)** Reactivation of GSNO- and H_2_O_2_-treated PAD4 by reducing agents. Rate of citrullination of BAEE by PAD4 [treated with GSNO (200 μM) or H_2_O_2_ (100 μM) for 10 min at 37°C] measured in presence of hTRX or DTT. **(B)** Combined effect of physiological reducing agents on PAD4 activation. PAD4 activity was measured in the presence of various concentrations of GSH containing sub-saturating (0.66 μM) and saturating concentrations of hTRX (5 μM).

The role of the oxidoreductase activity of hTRX was further evaluated by re-activating PAD4 after mild treatment with thiol-oxidizing (H_2_O_2_) and -nitrosylating (GSNO) agents (Figure 2A). Under both situations, hTRX revived PAD4 activity; but unlike control, only 50% (GSNO-treated PAD4) and 75% (H_2_O_2_-treated PAD4) of the activity revived by the *in vitro* reducing agent DTT (Figure 2A). Since GSH is a known co-activator of PADs, the combined effect of hTRX and GSH on PAD4 activity was also examined. PAD4 activity was measured in the presence of various concentrations of GSH containing sub-saturating (near *K*_d_ value) and saturating concentrations of hTRX (as determined in the previous section). As expected both hTRX (5 μM) and GSH (2 mM) individually showed activation of PAD4, but when used in combination there was only slight increase in maximum activity (Figure 2B).

### Kinetic Characterization of PAD4 Using hTRX as Reducing Agent

**Figure 3:**
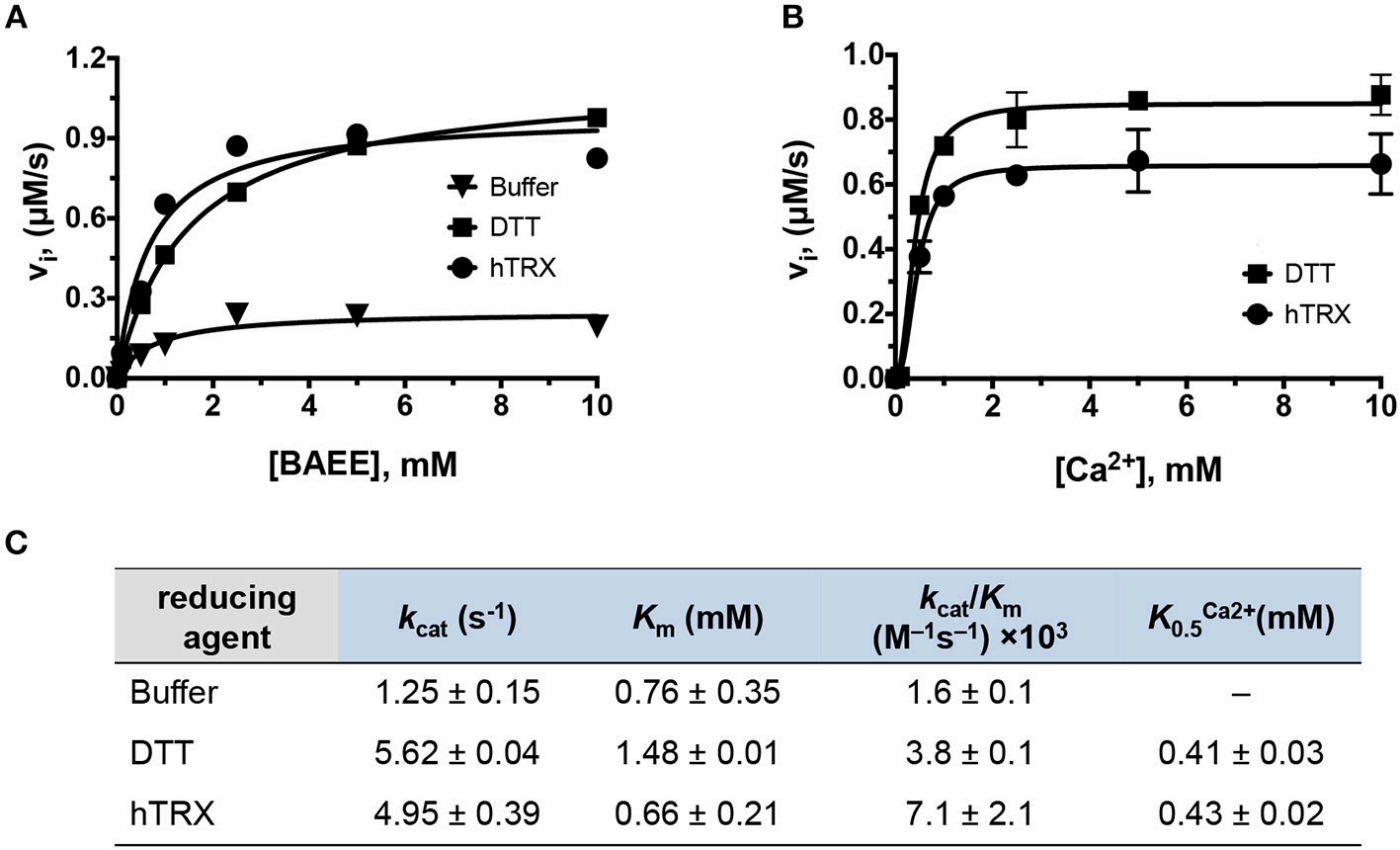
Kinetic characterization of PAD4. **(A)** Michaelis-Menten plots of PAD4 with various BAEE concentrations in presence of hTRX (5 μM), DTT (2 mM), and buffer as control. Refer to Figure S1 for PAD1, PAD2, and PAD3 data. **(B)** Calcium-dependence of PAD4 with hTRX and DTT as reducing agents. **(C)** Kinetic parameters deduced from **(A,B)**.

Next, the kinetic parameters of PAD4 for BAEE were determined in the presence of hTRX ($k_{\text{cat}}^{\text{hTRX}}$ = 5.0 ± 0.4 s^−1^; $K_{\text{m}}^{\text{hTRX}}=$ 0.7 ± 0.2 mM; $K_{0.5}^{\text{Ca}2+}$ = 0.43 ± 0.02 mM) and compared to the kinetic parameters obtained in presence of DTT ($k_{\text{cat}}^{\text{DTT}}$ = 5.62 ± 0.04 s^−1^; $K_{\text{m}}^{\text{DTT}}=$ 1.48 ± 0.01 mM; $K_{0.5}^{\text{Ca}2+}$ = 0.41 ± 0.03 mM) (Figures 3A–C). In both cases, all kinetic parameters including the Ca^2+^-dependence, were quite comparable (i.e., within one- to two-fold), suggesting hTRX could be the major PAD4 reducing agent under physiological conditions. In addition to PAD4, we also observed higher PAD1, PAD2, and PAD3 activity with buffer containing hTRX compared to buffer without any reducing agent, suggesting that the reducing activity of hTRX can activate all PAD isozymes (Figure S1).

### Impact of Redox-Activity of hTRX on PAD4 Activation

**Figure 4:**
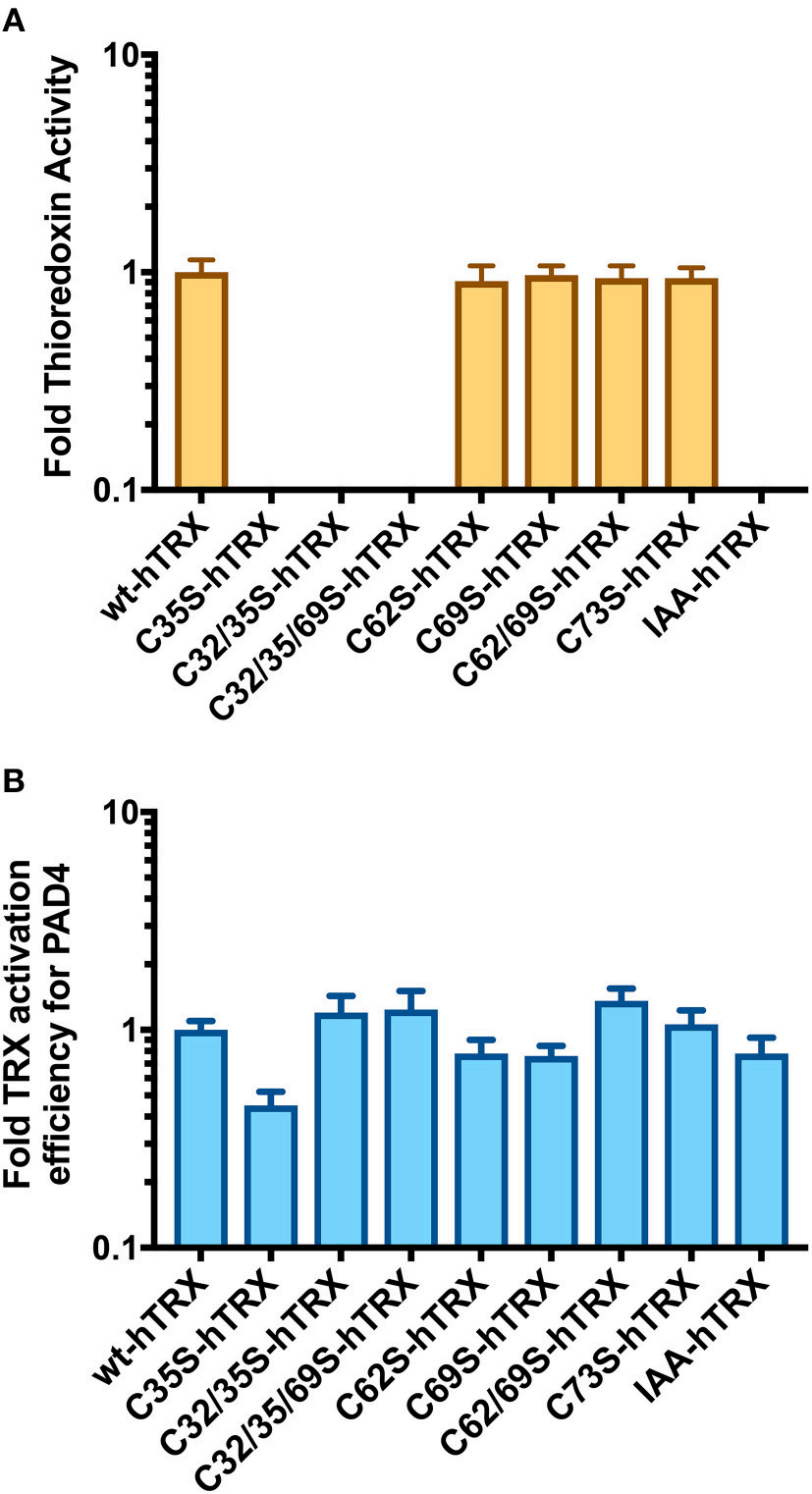
Activation of PAD4 in presence of hTRX variants. **(A)** Effect of hTRX mutations and IAA-treatment on its oxidoreductase activity. The hTRX activity was measured using a kit from Cayman Chemical Co. (cat# 20039). **(B)** Effect of various hTRX mutants on the catalytic efficiency of PAD4. Refer to Table 1 for the raw data. Fold change in thioredoxin activity or thioredoxin activation efficiency for PAD4 for the hTRX mutants are calculated with respect to wild-type hTRX.

To determine how hTRX activates PAD4, we first created various thioredoxin mutants and confirmed their oxidoreductase activity using a commercial assay kit. As expected, none of the hTRX active-site mutants, i.e., C35S, C32/35S, and C32/35/69S, were redox active (Figure 4A). Interestingly, these mutants were equally potent in activating PAD4 compared to wild type-hTRX (Figure 4B, Table 1). In addition, other redox-active TRX cysteine mutants (C62S, C69S, C62/69S, and C73S) behaved like wt hTRX and showed PAD4 activation suggesting that no individual cysteine residue is necessary for PAD4 activation (Table 1). To test this hypothesis, we created a thiol-free variant of TRX by chemically modifying all cysteine residues with iodoacetamide. Indeed, IAA-treated hTRX showed no redox activity, but it was found to be as efficient as that of other redox active hTRX variants in enhancing the rate of PAD4-catalyzed citrullination (Figures 4A,B).

**Table 1:** Activation of PAD4 by various hTRX variants.

**hTRX**	***V*_max_ (μM/s)**	***K*_d (app)_ (μM)**	**(*V*_max_/E_T_)/*K*_d (app)_ (M^−1^s^−1^) × 10^6^**
WT	0.83 ± 0.01	0.72 ± 0.05	5.7 ± 0.4
C35S	0.62 ± 0.02	1.17 ± 0.14	2.6 ± 0.4
C32/35S	0.61 ± 0.02	0.44 ± 0.08	7.0 ± 1.3
C32/35/69S	0.43 ± 0.02	0.30 ± 0.06	7.2 ± 1.5
C62S	1.03 ± 0.04	1.14 ± 0.14	4.5 ± 0.6
C69S	1.05 ± 0.05	1.38 ± 0.19	3.8 ± 0.4
C62/69S	0.96 ± 0.03	0.61 ± 0.07	7.9 ± 1.0
C73S	0.96 ± 0.04	0.78 ± 0.11	6.2 ± 0.9
IAA-hTRX	0.56 ± 0.02	0.62 ± 0.10	4.5 ± 0.7

*K_d(app)_, apparent binding affinity of PAD4 for hTRX variant*.

*(V_max_/E_T_)/K_d(app)_, pseudo activation-efficiency of hTRX variants*.

### hTRX Interaction With PAD4

**Figure 5:**
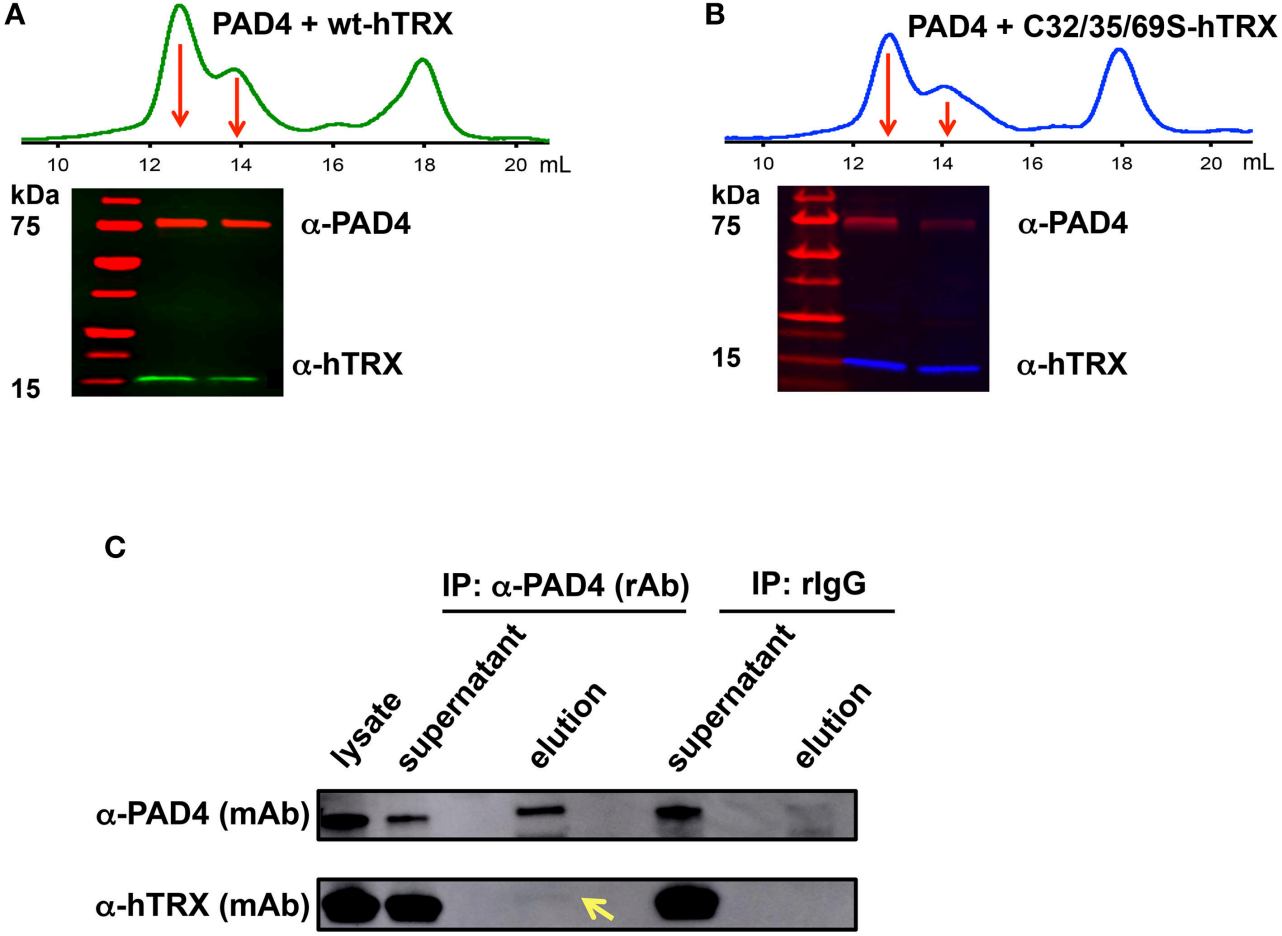
Interaction between PAD4 and hTRX. Size-exclusion chromatogram shows the elution profile of hTRX-PAD4 (**A**, green) and C32/35/69S-hTRX-PAD4 (**B**, blue) reaction mixture. The first and the second peak (marked with arrows) corresponding to dimeric and monomeric PAD4, respectively, were subjected to denaturing SDS-PAGE and the subsequent western blot of the PAD4 peak fractions shows the presence of the corresponding hTRX variant. Co-immunoprecipitation of hTRX with PAD4 **(C)**. Lysate from DMSO-differentiated HL60 cells was immunoprecipitated with anti-PAD4 (rAb) followed by western blot with anti-hTRX (mAb) and anti-PAD4 (mAb).

We then used size-exclusion chromatography and western blotting to investigate whether hTRX physically interacts with PAD4 (Figures 5A,B). The reaction mixture containing hTRX or the C32/35/69S-hTRX mutant and PAD4 yielded 3 major peaks after passing through the size-exclusion column. The first and second peaks represent dimeric and monomeric PAD4 respectively, and the last peak corresponds to hTRX (Figures 5A,B). Both PAD4 peaks were electro-transferred to a PVDF membrane and tested for the presence of hTRX using an anti-hTRX antibody. Samples from both redox-active hTRX and redox-inactive C32/35/69S-hTRX showed the presence of a corresponding hTRX band in the PAD4-containing lanes (Figures 5A,B), suggesting that these two proteins do interact in solution and that this interaction is not mediated though thiol-exchange.

Next, we determined whether hTRX interacts with PAD4 *in vivo* using a co-IP assay. IP experiments were performed using lysate of DMSO-differentiated HL60 cells that express higher levels of PAD4 (47) and anti-PAD4 (rabbit) antibody (Figure 5C). The presence of PAD4 and hTRX was evaluated in the input (lysate), unbound fractions (supernatant), and elution fractions (collected from the beads) using anti-PAD4 (mouse) and anti-hTRX (mouse) antibodies. The eluate from anti-PAD4 IP shows the presence of hTRX, confirming its interaction with PAD4 under cellular conditions (Figure 5C).

## Discussion

In recent decades, PAD-catalyzed citrullination has come into focus due to its role in various autoimmune diseases including RA. Although the precise cause of RA is unknown, it is commonly accepted that various environmental factors (e.g., smoking) trigger PAD activity to generate citrullinated proteins against which genetically susceptible individuals produce ACPAs (48–50). Since inflammation and oxidative stress are closely related pathophysiological processes, it has been proposed that oxidative stress is also correlated with RA pathogenesis (51, 52). In fact, several studies show that subjects with RA have high oxidative stress compared to healthy individuals (40, 53, 54). Mammalian cells have two major anti-oxidative defense mechanisms: reduced glutathione (GSH) and thioredoxin (hTRX). Although GSH is the major redox regulator, this system is impaired during oxidative stress and therefore RA patients show decreased (< 50% of healthy controls) levels of GSH (55–58). Despite lower GSH levels, Damgaard et al. showed that GSH is a major physiological co-regulator of PAD activity (34). By contrast, oxidative stress induces hTRX production during infection and inflammation, and acts as a chemoattractant for immune cells such as neutrophils, monocytes and T-cells (59, 60). Consequently, hTRX levels are elevated in serum, synovial fluid, and synovial tissues of RA patients (38–42). Here, we provide a potential link between high TRX levels and enhanced protein citrullination during RA.

Our data show that hTRX promotes PAD-catalyzed citrullination in a concentration-dependent manner (Figures 1A–D). Although hTRX is not a PAD substrate, it exhibits Michaelis-Menten-type kinetics indicating complex formation between these two proteins through stabilizing interactions (61, 62). Therefore, the TRX concentration that produces half—maximum PAD activation can be used as an estimate for binding affinity of hTRX for PAD (similar to the observed *K*_m_ for a substrate) (Table 1). Enhanced activation of PAD4 by DTT-treated hTRX (Figures 1A,B), and reactivation of GSNO and H_2_O_2_-treated PAD4 by hTRX is in accord with the fact that a reducing environment is an important requirement for PAD activity (Figure 2A) (34). In fact, saturating concentrations of reduced hTRX showed similar effects on PAD catalysis as that of DTT (Figures 2A, 3). Interestingly, the optimal hTRX concentration to activate PAD4 is 400-fold less than that of GSH (Figure 2B), but unlike GSH (34), hTRX does not impact the Ca^2+^-dependence of PAD4. Under physiological conditions, PAD4 activity is likely regulated by the balance of these two redox regulators and corresponding Ca^2+^-levels.

**Figure 6:**
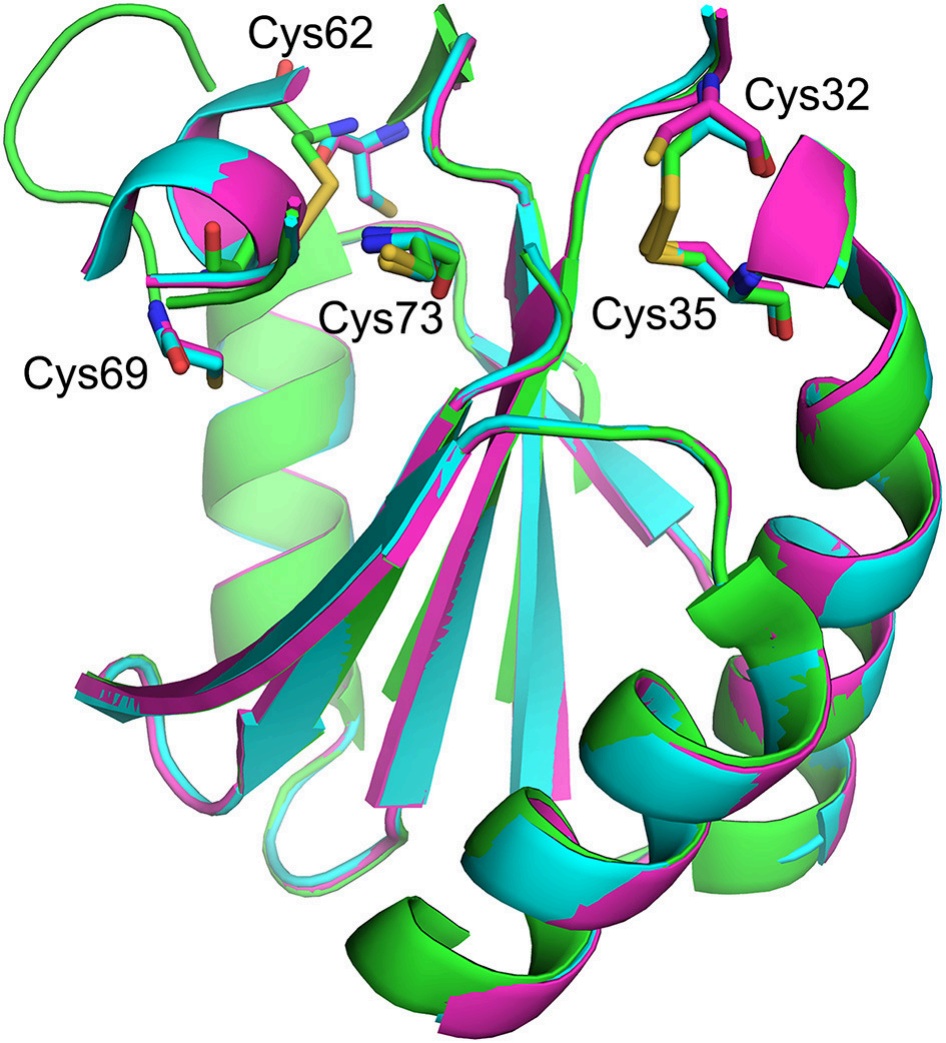
Structural overlay showing redox-active sites of hTRX in various oxidation states. Fully reduced (magenta, [PDB: 1ERT]), partially oxidized (blue, [PDB: 1ERV]), and fully oxidized (green, [PDB: 5DQY]).

TRX contains two redox-active sites (Cys 32 and Cys 35) and (Cys 62 and Cys 69). The former pair catalyzes most redox activity, whereas the latter redox active dithiol/disulfide pair (Cys 62 and Cys 69) is utilized under oxidizing conditions (Figure 6) (63). Therefore, converting two active-site cysteines (Cys 32 and Cys 35) to serines yields hTRX variants devoid of classical redox activity, while changing Cys 62 and Cys 69 to serines has no impact on redox activity (Figure 5A). Surprisingly, redox-inactive hTRX variants, i.e., C35S, C32/35S, and C32/35/69S activated PAD4 to catalyze citrullination suggesting that the redox activity of TRX is not essential for PAD4 activation (Figures 4A,B). To our knowledge there are only few prior studies featuring such TRX behavior (62, 64, 65). Liu et al. showed that TRX promotes ASK1 ubiquitination in a redox independent manner and that the association of TRX and ASK1 requires neither of the active site cysteines (Cys 32 or Cys 35) (64). Similarly, Huber et al. showed that active site cysteines are not required for TRX-dependent stimulation of DNA polymerase activity in *E. coli* but substitution or alkylation of these residues reduced the binding affinity by 60-fold indicating role for a TRX active site in protein-protein interactions (65). On the contrary, our data show minimal effects (one- to two-fold) on binding of hTRX to PAD4 upon substituting redox-active cysteines with serines (Table 1) suggesting that the activity-enhancing interactions may occur at residues other than redox-active cysteines. In fact, similar observations are reported for the hTRX-dependent activation of chloroplast NADP malate dehydrogenase and fructose-bisphosphatase where Cys32/35S substitution had no impact on its interaction or activation profile compared to wild type TRX. Although unusual, it is possible that electrostatic interactions between TRX and the target protein leads to formation of a non-covalent complex that influences the activity of the target protein (66). Indeed, we observed physical interactions between hTRX variants and PAD4 in *in vitro* studies (Figures 5A,B) as well as in DMSO-differentiated HL60 cells (Figure 5C). Furthermore, interactions between C32/35/69S-hTRX and PAD4 confirmed our prior inference from kinetic data that activity-enhancing interactions between hTRX and PAD4 occur at sites unaffected by mutation or chemical modification. In spite of several evidences that hTRX-mediated PAD activation does not require classical redox activity, DTT-treated hTRX showed enhanced activation of PAD4 compared to untreated hTRX. Such a difference can be attributed to change in binding of hTRX with PAD4 due to subtle conformational changes between reduced and oxidized forms of hTRX (Figure 6) (67, 68). In fact, the crystal structure of fully oxidized hTRX shows unraveling of a helix to form an extended loop (Figure 6, green). Since the binding affinity of oxidized and reduced hTRX was similar, one can only assume that the structural changes accompanying oxidation of hTRX makes the rate enhancing interactions less productive.

Generally, it is believed that PAD4-interacting proteins might promote its activity by lowering the Ca^2+^-dependence but this is not the case with hTRX (Figure 3B). Instead, non-covalently attached reduced hTRX likely provides localized high reduction potential near the active site that would prevent oxidation of the catalytic cysteine and facilitate the PAD4 catalysis. One such scenario could arise when hTRX and PAD share a substrate, for example, the transcription factor NF-κB. TRX is involved in the redox regulation of NF-κB, which is a known PAD substrate. In separate studies, Yosshida et al. showed that TRX accelerates the nuclear translocation of NF-kB (41) and Son et al. showed that citrullination of NF-κB enhances its nuclear localization (69). The present study provides a link between these processes and suggests that it could be hTRX mediated PAD4 catalyzed citrullination that enhances the nuclear localization of NF-κB.

**Figure 7:**
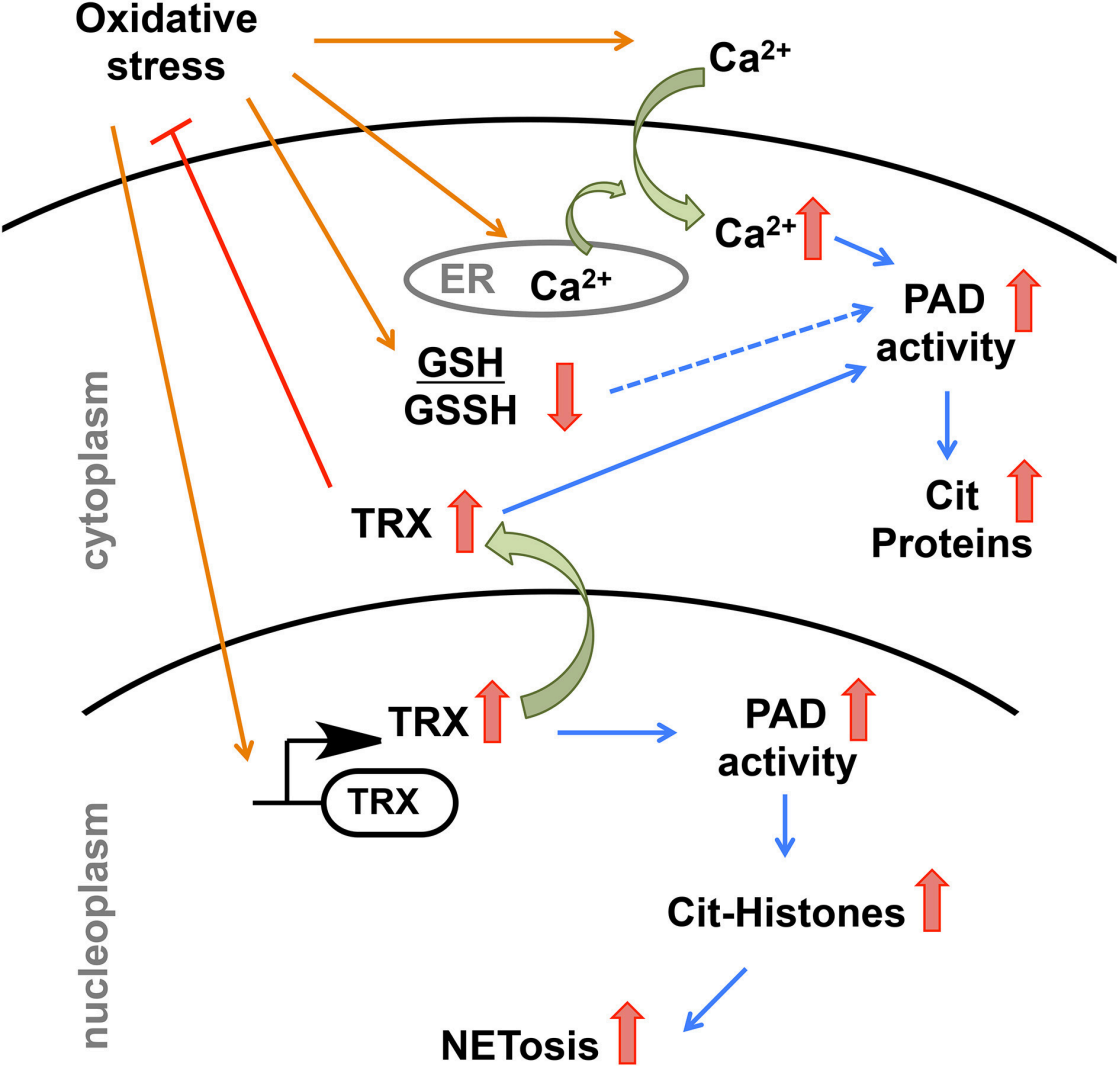
Model depicting possible regulation of PAD4 activity upon environmental stimuli that induces oxidative stress.

Based on our findings and the existing literature, we propose the following model to explain how environmental factors could trigger dysregulated PAD activity (Figure 7). Environmental factors that induce oxidative stress, such as smoking, pollution, and infections leads to higher expression of hTRX to combat increasing reactive oxygen species (59, 70, 71). Apart from hTRX, oxidative stress increases intracellular concentration of Ca^2+^ by promoting the influx of Ca^2+^ from the extracellular matrix and by releasing Ca^2+^ from reserves in the ER (72, 73). Thus, oxidative stress provides perfect conditions, i.e., elevated levels of hTRX and Ca^2+^, to trigger PAD activity in the cytosol as well as in the nucleus. In fact, H_2_O_2_ treated primary murine and human neutrophils showed enhanced PAD4-mediated histone citrullination (74, 75). Such aberrant PAD activity in the nucleus can cause NETosis, an event that would release active PADs into the extracellular matrix leading to uncontrolled citrullination and its pathogenic repercussions (Figure 7) (5–7). In summary, our data show that hTRX regulates PAD4 activity through a non-canonical mechanism that does not require its reducing activity; instead, non-covalent interactions are sufficient for thioredoxin mediated PAD activation.

## Author Contributions

MN and PT designed the study and analyzed data, and wrote the manuscript. MN performed the experiments. RT optimized PAD4 expression in HL60 cells.

### Conflict of Interest Statement

PT was a founder of Padlock Therapeutics which was acquired by Bristol Myers Squibb in 2016 and is entitled to payments if certain milestones are met. PT is a consultant for Celgene and Disarm Therapeutics. The remaining authors declare that the research was conducted in the absence of any commercial or financial relationships that could be construed as a potential conflict of interest.

## Abbreviations

hTRX: human thioredoxin
GSNO: *S*-nitrosoglutathione
RA: rheumatoid arthritis
PAD: protein arginine deiminase
ACPA: anti-citrullinated protein antibodies
NET: neutrophil extracellular trap
BAEE: *N*_α_-benzoyl arginine ethyl ester
PBS: phosphate buffered saline
BSA: bovine serum albumin.
