# Supplementary material for: Thioredoxin Modulates Protein Arginine Deiminase 4 (PAD4)-Catalyzed Citrullination
Source: Front Immunol. 2019 Feb 19;10:244. doi: 10.3389/fimmu.2019.00244 (PMC6396667; doi:10.3389/fimmu.2019.00244)
Supplement: Supplementary file 1 [file Data_Sheet_1.docx]

Supplementary Material

**Thioredoxin modulates Protein Arginine Deiminase 4 (PAD4)-catalyzed citrullination**

Mitesh Nagar^1,2,^ Ronak Tilvawala^1,2^ and Paul R. Thompson^1,2,*^

^1^Department of Biochemistry and Molecular Pharmacology, University of Massachusetts Medical School, 364 Plantation Street, Worcester, Massachusetts 01605, United States

^2^Program in Chemical Biology, University of Massachusetts Medical School, 364 Plantation Street, Worcester, Massachusetts 01605, United States

*** Correspondence:**
paul.thompson@umassmed.edu


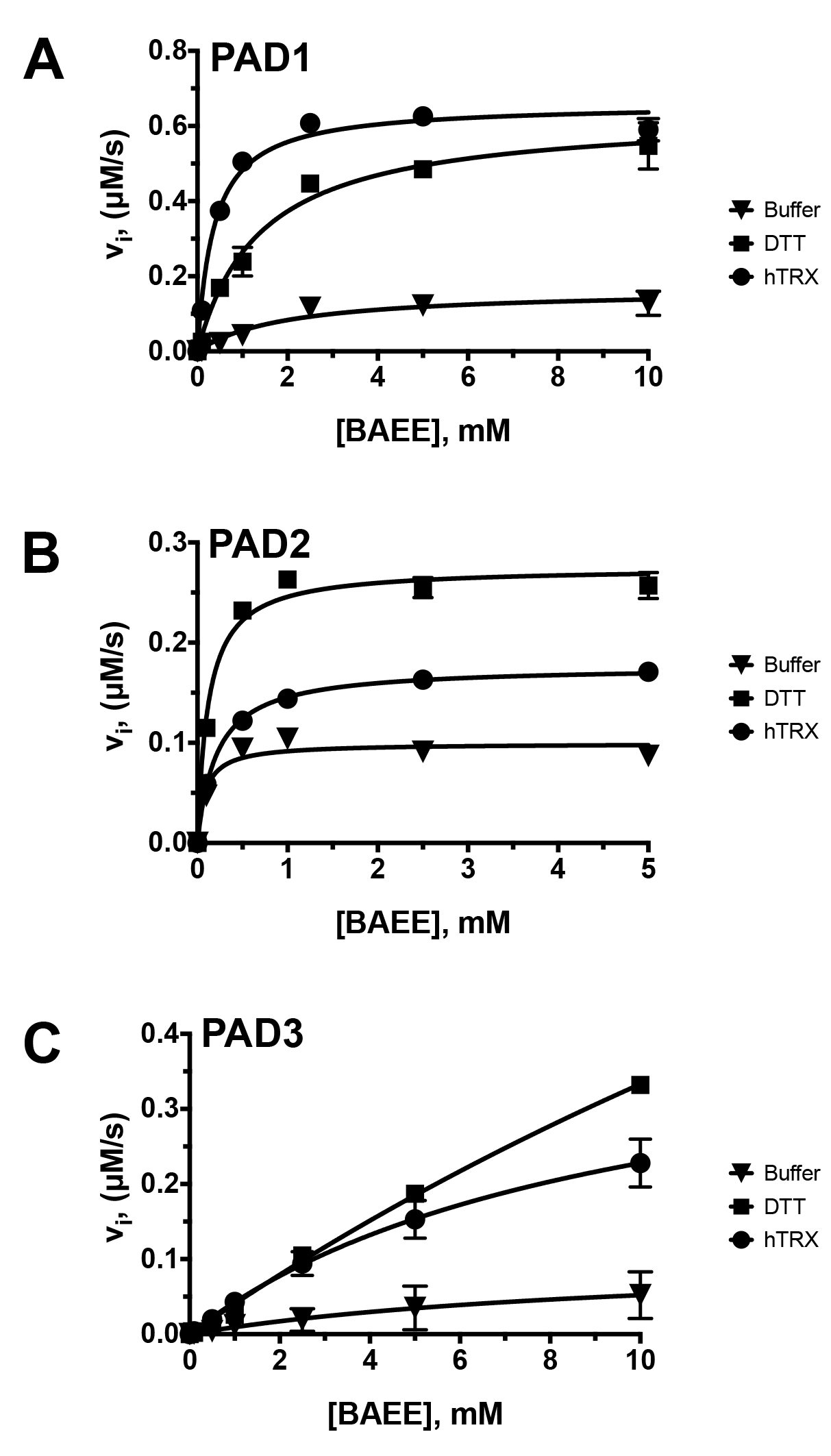


**Figure S1.**  Activation of various PAD isozymes. Michaelis-Menton plots of PAD1 **(A)**, PAD2 **(B)** and PAD3 **(C)** in presence of buffer (▼), DTT (2 mM, ■) and wt-hTRX (5 μM, ●). The final concentration of PAD1, PAD2 and PAD3 in the assays was 0.2, 1.0, and 1.0 μM respectively. As the enzymes were purified without reducing agents, PAD2 and PAD3 were found to be more prone to lose activity and therefore more enzyme was used in assay for better read outs.
